# Small cell cancer of the bladder: The Leon-Berard cancer centre experience

**DOI:** 10.4103/0970-1591.44255

**Published:** 2008

**Authors:** Nabil Ismaili, Fadi Elkarak, Pierre Etienne Heudel, Aude Flechon, Jean Pierre Droz

**Affiliations:** Medical Oncology, Centre Léon-Bérard, 28, Rue Laennec, Lyon-69373 - France

**Keywords:** Bladder, cancer, small cell

## Abstract

**Background:**

Small cell bladder carcinoma is an uncommon tumor. In this retrospective study we report our experience dealing with this disease at the Leon-Berard Cancer Centre.

**Materials and Methods:**

We retrospectively analyzed various characteristics of small cell bladder carcinoma: patient demographics, histological diagnosis, disease stage, treatment effects and outcome, in 14 non-metastatic small cell bladder carcinoma patients treated at our institution between 1995 and 2006.

**Results:**

The mean age at diagnosis was 60 years (range, 45-77). All patients were male. Seventy-five per cent were smokers. All had locally advanced disease. Ten patients (71.4%) were treated by cystoprostatectomy and bilateral pelvic lymph node resection, one by cystoprostatectomy alone. Two patients received neoadjuvant chemotherapy and four received adjuvant chemotherapy. One patient was treated by radiotherapy with concomitant cisplatin after transurethral resection of bladder tumor (TURBT). One patient refused surgery and was treated by chemotherapy alone. One patient was lost to follow-up after TURBT. After 49-month median follow-up, 12 patients had relapsed. Disease-free survival was 5.7 months. The most frequent sites of relapse were the retroperitoneal lymph node (seven patients) and the liver (three patients). Nine patients died of metastasis. Median overall survival was 29.5 months. Survival probability at two years was 58%. Median overall survival was 34 months in the mixed small carcinoma group, as compared with 9.5 months in the pure small cell carcinoma group (*P*=0.01). Mean overall survival was 27.2 months for all patients and 38.6 months for patients treated with cystectomy and adjuvant chemotherapy.

**Conclusion:**

To date, the optimal treatment for locally advanced small cell bladder carcinoma is not clear. Cystectomy with neoadjuvant or adjuvant chemotherapy appears as a viable option.

## INTRODUCTION

Whereas small cell cancer is a common histological variant accounting for 14% of all primitive cancers arising from the lung,[1] small cell bladder carcinoma is extremely rare and accounts for less than 1% of all cancers arising from the bladder.[2,3] To our knowledge, only 550 cases have been reported up to 2007. [4–10] This cancer is one of the rare extra-pulmonary localizations of small cell carcinomas. The first case was described in 1981 by Cramer et al.[11] Small cell bladder carcinomas are neuroendocrine epithelial tumors associated with a more aggressive behavior and poorer prognosis than transitional cell bladder carcinoma, and mostly diagnosed at advanced stages. Because of the rarity of the disease, no standard treatment has yet been proposed. For muscle-invasive disease, cystectomy, either alone or in combination with chemotherapy and/or radiotherapy, may be proposed. We conducted a retrospective analysis of all cases of muscle-invasive non-metastatic bladder cancer treated at the Leon-Berard Cancer Centre over a 12-year period.

## MATERIALS AND METHODS

### Identification of patients

We retrospectively searched the files of all patients with locally advanced or metastatic bladder carcinoma treated at the Leon-Berard Cancer Centre between January 1995 and December 2006 to identify patients with small cell disease. Patients were considered to have small cell bladder carcinoma if pathological examination of their tumor revealed the presence of any small cell component.

### Data collection and follow-up

Patient medical records were retrospectively searched for demographics, clinical stage, histological findings, treatment and outcome. Radiological, pathological and surgical reports were reviewed to determine the stage of the disease at the time of cystectomy by using the 2002 TNM classification for genitourinary tumors.[12] Data about treatment, notably surgery, chemotherapy and radiotherapy, were extracted from patient medical records. The date and site of recurrence and, if applicable, the date and cause of death were also considered.

### Statistical analysis

Overall survival and recurrence-free survival were analyzed statistically in all patients. Overall survival was calculated from the date of diagnosis to the date of death or to the date of last follow-up. Time to recurrence was calculated from the date of surgery to the date of first documented relapse or to the date of last follow-up. We retrospectively compared disease-free survival and overall survival between the pure small cell carcinoma group (n=5) and mixed small cell carcinoma group (n=9). The Kaplan-Meier method was used to calculate median time to recurrence and median overall survival. The log-rank test was used to evaluate the differences between the groups.

## RESULTS

Between 1995 and 2006, 911 patients with locally advanced or metastatic bladder cancer were treated at the Léon-Bérard Cancer Centre. Only 17 patients had small cell histology, of whom 14 had muscle-invasive non-metastatic disease. Table 1 summarizes the characteristics and clinico-pathological findings of these 14 patients. Mean age at diagnosis was 60 years (range, 45-77 years). All patients were male. Seventy-five per cent of the patients were smokers. Nine patients had both transitional cell and small cell histology, while five had exclusive small cell histology. All patients had a locally advanced T2 disease on transurethral resection of the bladder tumor (TURBT). Four patients had clinical stage II, five had clinical stage III and five had clinical stage IV with no distant metastasis at initial staging. Ten patients (71.4%) were treated by cystoprostatectomy and bilateral pelvic lymph node resection, one by cystoprostatectomy alone. Two patients received neoadjuvant chemotherapy and four received adjuvant chemotherapy. Chemotherapy was a combination of etoposide and cisplatin. One patient was treated by radiotherapy with concomitant cisplatin after TURBT. One was treated by chemotherapy alone because he refused surgery. Finally, one patient was lost to follow-up after TURBT.

**Table 1:** Demographic and histopathological characteristics

Characteristics	No. of patients (%)
Total	14
Age at diagnosis
Mean	60.5
Range	45-78
Gender
Male	14 (100)
Female	0
Smoking history
Present	11 (78.5)
Absent	3 (21.5)
Histological finding
SCC only	5 (35.7)
SCC and TCC	9 (64.3)
Stage in initial management
Stage II	4 (28.6)
Stage III	5 (35.7)
Stage IV (M0)	5 (35.7)

**Figure 1:**
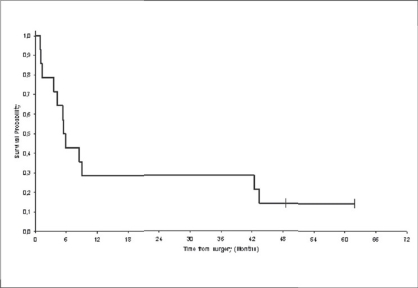
Disease-Free Survival (DFS): The delay of disease-free survival was calculated by the date of surgery until the date of revealing of a progress or until the date of death, or until the date of last news. The median duration of survival without progress is presented as well as the median follow-up and the rate of survival without progress in two years. N = 14 (12 events); Survival median: 5.7 months; Survival probability at two years: 28%

**Figure 2:**
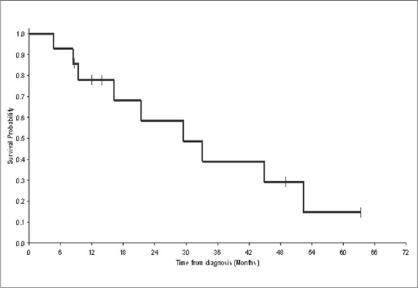
Overall survival (OS): The delay of overall survival was calculated by the date of diagnosis until the death or until the date of last news. The median duration of global survival is presented as well as the median follow-up and the rate of global survival in two years. N = 14 (nine events); Survival median: 29.5 months; Survival probability at two years: 58%

**Figure 3:**
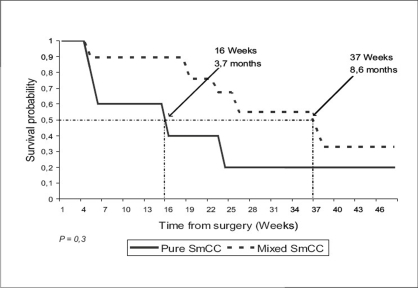
Disease-free survival in patients with pure neuroendocrine tumors of bladder vs. tumors with mixed histology: The median duration of disease-free survival (indicated by the dotted lines) was 8.6 months in the mixed small cell carcinoma (SmCC) group, as compared with 3.7 months in the pure group (*P*=0.3)

**Figure 4:**
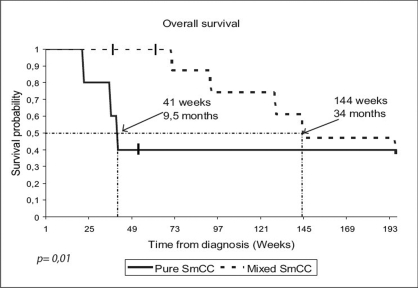
Overall survival in patients with pure neuroendocrine tumors of bladder vs. tumors with mixed histology: The median duration of survival (indicated by the dotted lines) was 34 months in the mixed small cell carcinoma (SmCC) group, as compared with 9.5 months in the pure SmCC group (*P* = 0.01)

After 49-month median follow-up, 12 patients had relapsed. Median disease-free survival was 5.7 months [Figure 1]. The most frequent sites of relapse were the retroperitoneal lymph nodes (seven patients) and the liver (three patients) [Table 2]. Nine patients died of metastatic disease. Median overall survival was 29.5 months [Figure 2]. Survival probability at two years was 58%. The median duration of disease-free survival was 8.6 months in the mixed small cell carcinoma group, as compared with 3.7 months in the pure small cell carcinoma group (*P* = 0.3) [Figure 3]. The median duration of survival was 34 months in the mixed small cell carcinoma group, as compared with 9.5 months in the pure small cell carcinoma group (*P* = 0.01) [Figure 4]. Mean overall survival was 27.2 months for the whole group of patients and 38.6 months for patients treated with cystectomy and adjuvant chemotherapy. The survival of the patient treated by chemotherapy alone was 9.2 months. The mean survival of the five patients treated by surgery alone was 22.5 months. Two patients are still alive. All the five patients have received the chemotherapy after progression. The patients treated by concurrent chemoradiotherapy were still alive without disease after 49.7 months.

**Table 2:** Sites of recurrence

Sites of recurrence	No. of patients
Local recurrence	1
Nodal recurrence	4
Metastatical recurrence
Nodal and liver	2
Brain	1
Nodal and lung	1
Liver and bone	1

## DISCUSSION

Small cell bladder carcinoma was initially described in 1981 by Cramer *et al*.[11] On histology, the small cell component appears as small cells with scant cytoplasm, ill-defined cell borders, finely granular nuclear chromatin, and reduced or absent nucleoli. Cells are round or oval, and the mitotic count is high. Immunostaining analysis shows that cells express markers of neuroendocrine differentiation, including chromogranin A, neuron-specific enolase, neural adhesion molecule and synaptophysin.

Primary small cell carcinoma of the bladder is a rare disease that accounts for less than 1% of all bladder cancers.[2,3] The relatively high incidence of 1.8% reported here might reflect a bias due to the fact that our centre mostly recruits patients with metastatic bladder cancer. We found that most small cell bladder cancers (64%) were admixed with transitional cell carcinoma. Other teams have shown a higher incidence of pure small cell carcinoma.[10]

Because small cell bladder carcinoma is rare, and in the absence of randomized controlled trials, there is no standard treatment for muscle-invasive non-metastatic disease. In a multi-institutional review of 64 patients, a multivariate analysis has indicated that neither chemotherapy, nor radiation, nor surgery has any impact on overall survival.[7] The poor prognosis of patients treated by radical cystoprostatectomy alone reported by Sved *et al*.,[8] supports the use of combination modality treatments. In our series, the mean survival of the five patients treated by surgery alone was 22.5 months. A retrospective study of 46 patients treated at the M. D. Anderson Cancer Centre has reported a five-year survival of 78% for patients receiving neoadjuvant chemotherapy followed by cystectomy, versus 36% for patients undergoing cystectomy alone.[9] In the Mayo Clinic Study, the authors have proposed radical cystectomy for patients with locally advanced disease and adjuvant treatment for patients with Stage III and VI (M0) disease.[10] The results obtained in our series are in concordance with these previous results and show a 38.6-month mean survival for patients treated with radical cystectomy and adjuvant chemotherapy. The protocol of chemotherapy recommended by the M. D. Anderson Cancer Center was the associations used in the neuroendocrine tumors for both pure small cell carcinoma and mixed small cell carcinoma of the bladder.[13] Bastus *et al*., have reported on a series of five patients with locally advanced disease treated with sequential chemoradiotherapy. Four patients have had long-term disease-free survival and overall survival.[14] In another study, the authors have expressed a realistic optimism and acknowledged a potential for long-term survival in patients with limited stage small cell cancer of the bladder treated with a combination of chemotherapy and local radiotherapy; the actuarial survival rate was 70% at two years and 44% at five years.[15] In our series, the patient treated with concurrent chemoradiotherapy was still alive without disease after 49.7 months. The most common site of relapse in our study was the retroperitoneal lymph nodes (50% of the patients), suggesting that retroperitoneal lymph node irradiation after radical cystectomy might possibly improve disease-free survival. Of the five patients with retroperitoneal lymph node disease, three relapsed in the retroperitoneum, one in the retroperitoneum and the liver, and one in the lung. Finally, we found that pure small cell carcinoma has poorer outcome than mixed small cell carcinoma of the bladder. The median duration of survival was 34 months in the mixed small cell carcinoma group, as compared with 9.5 months in the pure small cell carcinoma group (*P*=0.01) [Figure 4]. In one series, mixed histologies tended to do better than pure neuroendocrine tumors, although this did not attain statistical significance (*P*=0.064).[16]

## CONCLUSION

In the absence of prospective study, the best treatment for small cell bladder cancer cannot be established for certain. From our study and from the literature, it appears that combined treatment is necessary for achieving long-term recurrence-free and overall survival. Cystectomy with neoadjuvant or adjuvant chemotherapy appears to be a logical option. The association of chemotherapy and radiotherapy should also be considered. In addition, the pure small cell carcinoma appears to have poorer outcome than the mixed small cell carcinoma of the bladder.
